# Supplementary material for: Genome-wide CRISPR screening reveals genetic modifiers of mutant EGFR dependence in human NSCLC
Source: eLife. 2019 Nov 19;8:e50223. doi: 10.7554/eLife.50223 (PMC6927754; doi:10.7554/eLife.50223)
Supplement: Supplementary file 3. [file elife-50223-supp3.docx]

| **Key Resources Table** | | | | |
| --- | --- | --- | --- | --- |
| **Reagent type (species) or resource** | **Designation** | **Source or reference** | **Identifiers** | **Additional information** |
| Strain, strain background (*E. coli*) | Stbl3 | Invitrogen | Cat#:C737303 | Chemically competent *E. coli* for cloning |
| Cell line (*Homo sapiens*) | HEK293T | ATCC | Cat#:CRL-11268;  RRID:CVCL_1926 | For lentivirus production |
| Cell line (*Homo sapiens*) | HEK293A | Invitrogen | Cat#:R70507;  RRID:CVCL_6910 |  |
| Cell line (*Homo sapiens*) | HEK293A-GTIIC-GFP-Cas9 | This paper |  | YAP reporter cell line |
| Cell line (*Homo sapiens*) | HCC827 | CCLE | ATCC:CRL-2868; CCLE:HCC827_LUNG;  RRID:CVCL_2063 |  |
| Cell line (*Homo sapiens*) | NCI-H3255 | CCLE | ATCC:CRL-2882; CCLE:NCIH3255_LUNG;  RRID:CVCL_6831 |  |
| Cell line (*Homo sapiens*) | HCC4006 | CCLE | ATCC:CRL-2871; CCLE:HCC4006_LUNG;  RRID:CVCL_1269 |  |
| Cell line (*Homo sapiens*) | PC9 | CCLE | CCLE: PC9_LUNG;  RRID:CVCL_B260 |  |
| Cell line (*Homo sapiens*) | NCI-H1975 | CCLE | ATCC:CRL-5908; CCLE:NCIH1975_LUNG;  RRID:CVCL_1511 |  |
| Cell line (*Homo sapiens*) | A549 | CCLE | ATCC:CCL-185; CCLE:A549_LUNG;  RRID:CVCL_0023 |  |
| Cell line (*Homo sapiens*) | NCI-H1299 | CCLE | ATCC:CRL-5803; CCLE:NCIH1299_LUNG;  RRID:CVCL_0060 |  |
| Cell line (*Homo sapiens*) | NCI-H460 | CCLE | ATCC:HTB-177; CCLE:NCIH460_LUNG;  RRID:CVCL_0459 |  |
| Cell line (*Homo sapiens*) | BEAS-2B | ATCC | ATCC:CRL-9609;  RRID:CVCL_0168 |  |
| Antibody | Rabbit polyclonal anti-PRK2/PKN2 | Cell Signaling Technology | Cat#:2612  RRID:AB_2167753 | WB (1:1000) |
| Antibody | Rabbit monoclonal anti-YAP (D8H1X) | Cell Signaling Technology | Cat#:14074  RRID:AB_2650491 | WB (1:1000) |
| Antibody | Rabbit monoclonal anti-YAP/TAZ (D24E4) | Cell Signaling Technology | Cat#:8418  RRID:AB_10950494 | WB (1:1000) |
| Antibody | Rabbit polyclonal anti-phospho-YAP (Ser127) | Cell Signaling Technology | Cat#:4911  RRID:AB_2218913 | WB (1:1000) |
| Antibody | Rabbit monoclonal anti-phospho-EGFR (Tyr1068) | Cell Signaling Technology | Cat#:3777  RRID:AB_2096270 | WB (1:1000) |
| Antibody | Rabbit monoclonal anti-EGFR (D38B1) | Cell Signaling Technology | Cat#:4267  RRID:AB_2246311 | WB (1:20000) |
| Antibody | Rabbit monoclonal anti-phospho-Akt (Ser473) (193H12) | Cell Signaling Technology | Cat#:4058  RRID:AB_331168 | WB (1:1000) |
| Antibody | Rabbit polyclonal anti-Akt | Cell Signaling Technology | Cat#:9272  RRID:AB_329827 | WB (1:5000) |
| Antibody | Rabbit polyclonal anti-phospho-Erk1/2 (Thr202/Tyr204) | Cell Signaling Technology | Cat#:9101  RRID:AB_331646 | WB (1:1000) |
| Antibody | Rabbit polyclonal anti-Erk1/2 | Cell Signaling Technology | Cat#:9102  RRID:AB_330744 | WB (1:5000) |
| Antibody | Rabbit polyclonal anti-ARIH2/TRIAD1 | Cell Signaling Technology | Cat#:13689  RRID:AB_2798293 | WB (1:1000) |
| Antibody | Rabbit polyclonal anti-BTAF1 | Cell Signaling Technology | Cat#:2637  RRID:AB_11139999 | WB (1:1000) |
| Antibody | Rabbit monoclonal anti-GNAQ (D5V1B) | Cell Signaling Technology | Cat#:14373  RRID:AB_2665457 | WB (1:1000) |
| Antibody | Rabbit monoclonal anti-HSP90 (C45G5) | Cell Signaling Technology | Cat#:4877  RRID:AB_2233307 | WB (1:5000) |
| Antibody | Rabbit monoclonal anti-ALDOA (D73H4) | Cell Signaling Technology | Cat#:8060  RRID:AB_2797635 | WB (1:5000) |
| Antibody | Rabbit monoclonal anti-METAP2 (D3I1H) | Cell Signaling Technology | Cat#:12547  RRID:AB_2797951 | WB (1:1000) |
| Antibody | Rabbit monoclonal anti-GAPDH (14C10) | Cell Signaling Technology | Cat#:2118  RRID:AB_561053 | WB (1:5000) |
| Antibody | Mouse monoclonal anti-α-Tubulin (Clone B-5-1-2) | Sigma-Aldrich | Cat#:T6074  RRID:AB_477582 | WB (1:20000) |
| Antibody | Mouse monoclonal anti-β-Actin (Clone AC-15) | Sigma-Aldrich | Cat#:A1978  RRID:AB_476692 | WB (1:20000) |
| Antibody | Rabbit monoclonal anti-GNB2 (EPR3261Y) | Abcam | Cat#:ab81272  RRID:AB_1640549 | WB (1:1000) |
| Antibody | Rabbit polyclonal anti-RIC8A | Abcam | Cat#:ab97808  RRID:AB_10696254 | WB (1:1000) |
| Antibody | Rabbit monoclonal anti-USP22 (EPR18945) | Abcam | Cat#:ab195289  RRID:AB_2801585 | WB (1:1000) |
| Antibody | Rabbit monoclonal anti-CUL5 (EPR14725) | Abcam | Cat#:ab184177 | WB (1:1000) |
| Antibody | Rabbit monoclonal anti-RNF7 (EPR12001) | Abcam | Cat#:ab181986 | WB (1:1000) |
| Antibody | Rabbit polyclonal anti-PDCD10 | Abcam | Cat#:ab180706 | WB (1:1000) |
| Antibody | Rabbit polyclonal anti-KCTD5 | Proteintech | Cat#:15553-1-AP  RRID:AB_2132155 | WB (1:1000) |
| Antibody | Rabbit polyclonal anti-PSAT1 | Proteintech | Cat#:10501-1-AP  RRID:AB_2172597 | WB (1:5000) |
| Antibody | Goat Anti-Rabbit IgG Antibody, (H+L) HRP conjugate | Millipore Sigma | Cat#:AP307P  RRID:AB_11212848 | WB (1:5000) |
| Antibody | Goat Anti-Mouse IgG Antibody, (H+L) HRP conjugate | Millipore Sigma | Cat#:AP308P  RRID:AB_11215796 | WB (1:5000) |
| Commercial assay or kit | CellTiter-Glo Luminescent Cell Viablity Assay | Promega | Cat#:G7572 |  |
| Commercial assay or kit | Caspase-Glo 3/7 Assay System | Promega | Cat#:G8092 |  |
| Commercial assay or kit | DC Protein Assay Kit II | Bio-Rad | Cat#:5000112 |  |
| Commercial assay or kit | Click-iT Protein Reaction Buffer Kit | Thermo Fisher Scientific | Cat#:C10276 |  |
| Commercial assay or kit | RNeasy Plus Mini Kit | Qiagen | Cat#:74134 |  |
| Commercial assay or kit | TaqMan Reverse Transcription Reagents | Thermo Fisher Scientific | Cat#:N8080234 |  |
| Commercial assay or kit | TaqMan Fast Advanced Master Mix | Thermo Fisher Scientific | Cat#:4444557 |  |
| Commercial assay or kit | Q5 High-Fidelity 2X Master Mix | NEB | Cat#:M0492S |  |
| Commercial assay or kit | QIAquick PCR Purification Kit | Qiagen | Cat#:28104 |  |
| Commercial assay or kit | QIAquick Gel Extraction Kit | Qiagen | Cat#:28704 |  |
| Commercial assay or kit | QIAprep Spin Miniprep Kit | Qiagen | Cat#:27104 |  |
| Commercial assay or kit | QIAamp DNA Blood Maxi Kit | Qiagen | Cat#:51194 |  |
| Commercial assay or kit | TUBE2 agarose beads | LifeSensors | Cat#:UM402 |  |
| Software, algorithm | ImageJ | NIH | <https://imagej.nih.gov/ij/>;  RRID:SCR_003070 |  |
| Software, algorithm | GraphPad Prism Version 8 | GraphPad | <https://www.graphpad.com/scientific-software/prism/>;  RRID:SCR_002798 |  |
| Software, algorithm | FlowJo Version 10 | FlowJo | <https://www.flowjo.com/solutions/flowjo/downloads>;  RRID:SCR_008520 |  |
