# Supplementary material for: Genome-wide CRISPR screening reveals genetic modifiers of mutant EGFR dependence in human NSCLC
Source: eLife. 2019 Nov 19;8:e50223. doi: 10.7554/eLife.50223 (PMC6927754; doi:10.7554/eLife.50223)
Supplement: Transparent reporting form [file elife-50223-transrepform.pdf]

1st Floor
24 Hills Road
Cambridge CB2 1JP, UK

P 01223 855340
W elifesciences.org
T @elife

# *eLife's* transparent reporting form

We encourage authors to provide detailed information *within their submission* to facilitate the interpretation and replication of experiments. Authors can upload supporting documentation to indicate the use of appropriate reporting guidelines for health-related research (see EQUATOR Network), life science research (see the BioSharing Information Resource), or the ARRIVE guidelines for reporting work involving animal research. Where applicable, authors should refer to any relevant reporting standards documents in this form.

If you have any questions, please consult our Journal Policies and/or contact us: editorial@elifesciences.org.

**Sample-size estimation**

- You should state whether an appropriate sample size was computed when the study was being designed
- You should state the statistical method of sample size computation and any required assumptions
- If no explicit power analysis was used, you should describe how you decided what sample (replicate) size (number) to use

Please outline where this information can be found within the submission (e.g., sections or figure legends), or explain why this information doesn't apply to your submission:

| |
|---|
| For genome-wide CRISPR screen, sgRNA library was transduced at a multiplicity of infection (MOI) of 0.5, aiming for coverage of, on average, 1000 cells per sgRNA reagent. For other experiments, sample size was not pre-determined with any statistical test. Sufficient sample sizes were estimated based on previous experiments with the same system. |

**Replicates**

- You should report how often each experiment was performed
- You should include a definition of biological versus technical replication
- The data obtained should be provided and sufficient information should be provided to indicate the number of independent biological and/or technical replicates
- If you encountered any outliers, you should describe how these were handled
- Criteria for exclusion/inclusion of data should be clearly stated
- High-throughput sequence data should be uploaded before submission, with a private link for reviewers provided (these are available from both GEO and ArrayExpress)

Please outline where this information can be found within the submission (e.g., sections or figure legends), or explain why this information doesn't apply to your submission:

| |
|---|
| Replicates information can be found in the figure legends. For CRISPR screen described in this study, hits selection and detailed sgRNA information were provided in Supplementary File 1 and Supplementary File 2. The mass spectrometry proteomics data have been deposited to the ProteomeXchange Consortium via the PRIDE partner repository with the dataset identifier PXD014198. |

eLife Sciences Publications, Ltd is a limited liability non-profit non-stock corporation incorporated in the State of Delaware, USA, with company number 5030732, and is registered in the UK with company number FC030576 and branch number BR015634 at the address 1st Floor, 24 Hills Road, Cambridge CB2 1JP | August 2014

**Statistical reporting**

- Statistical analysis methods should be described and justified
- Raw data should be presented in figures whenever informative to do so (typically when N per group is less than 10)
- For each experiment, you should identify the statistical tests used, exact values of N, definitions of center, methods of multiple test correction, and dispersion and precision measures (e.g., mean, median, SD, SEM, confidence intervals; and, for the major substantive results, a measure of effect size (e.g., Pearson's r, Cohen's d)
- Report exact p-values wherever possible alongside the summary statistics and 95% confidence intervals. These should be reported for all key questions and not only when the p-value is less than 0.05.

Please outline where this information can be found within the submission (e.g., sections or figure legends), or explain why this information doesn't apply to your submission:

Statistical information can be found in the figure legends. Unless otherwise noted, data are presented as the means ± SD. For all graphs, data are presented relative to their respective controls. Statistical analyses were performed using GraphPad Prism 7 (Graphpad software Inc.). Significance was determined by a two-tailed Student's *t* test or ordinary two-way ANOVA, denoted within each figure panel and respective figure legends. $P < 0.05$ was considered to be statistically significant. * $P < 0.05$, ** $P < 0.01$, *** $P < 0.001$, **** $P < 0.0001$; ns, not significant.

(For large datasets, or papers with a very large number of statistical tests, you may upload a single table file with tests, Ns, etc., with reference to sections in the manuscript.)

**Group allocation**

- Indicate how samples were allocated into experimental groups (in the case of clinical studies, please specify allocation to treatment method); if randomization was used, please also state if restricted randomization was applied
- Indicate if masking was used during group allocation, data collection and/or data analysis

Please outline where this information can be found within the submission (e.g., sections or figure legends), or explain why this information doesn't apply to your submission:

Mice were enrolled in the study once tumors had reached approximately 200 $mm^3$ in size (day 13 post-implantation), and were randomly assigned to receive either vehicle or erlotinib (LC Laboratories; 10 mg/kg) (compound formulation 50% Dexolve-7 (Generic SBECD) and 50% of 0.1 M Tartaric Acid) once daily by oral gavage for the duration of the study. This information can be found in the Methods section.

**Additional data files ("source data")**

- We encourage you to upload relevant additional data files, such as numerical data that are represented as a graph in a figure, or as a summary table
- Where provided, these should be in the most useful format, and they can be uploaded as "Source data" files linked to a main figure or table
- Include model definition files including the full list of parameters used
- Include code used for data analysis (e.g., R, MatLab)
- Avoid stating that data files are "available upon request"

Please indicate the figures or tables for which source data files have been provided:

CRISPR-Cas9 screen data were summarized in Supplementary File 1 and Supplementary File 2 to support Figure 1.

eLife Sciences Publications, Ltd is a limited liability non-profit non-stock corporation incorporated in the State of Delaware, USA, with company number 5030732, and is registered in the UK with company number FC030576 and branch number BR015634 at the address 1st Floor, 24 Hills Road, Cambridge CB2 1JP | August 2014
